# A Journey towards Resilience: Coping Strategies Adopted by Parents with Children Having Autism Spectrum Disorder in Northeast Malaysia

**DOI:** 10.3390/ijerph19042458

**Published:** 2022-02-21

**Authors:** Wan Natrah Wan Yaacob, Lili Husniati Yaacob, Maryam Mohd Zulkifli, Rosediani Muhamad

**Affiliations:** Department of Family Medicine, School of Medical Sciences, Universiti Sains Malaysia, Kota Bharu 16150, Malaysia; natrah.64@gmail.com (W.N.W.Y.); rosesyam@usm.my (R.M.)

**Keywords:** autism, parents, coping, resilience

## Abstract

Background: The prevalence of Autism Spectrum Disorder (ASD) has been increasing for the past two decades. Parents with autism have been known to be at risk of psychological distress and maladaptation, but many parents were able to overcome this adversity and lead to a good quality of life. Methods: In-depth interviews were conducted among 21 parents of children with ASD. Interviews were transcribed and evaluated using thematic analysis. Results: The analysis discovered three main themes: acceptance and positive outlook, reaching for helping hands, and understanding autism and finding its solutions. Conclusions: Although the findings cannot be generalised to other populations with ASD, this study provides a detailed perspective on their unique coping strategies. Insights gained from this study could help health care providers, authorities, and communities to address a specific need and able to advocate relevant support measures to assist them.

## 1. Introduction

The Autism spectrum disorder (ASD) is a combination of qualitative impairments in reciprocal social interaction, communication, repetitive, restricted, and stereotyped patterns of behavior [1]. The American Psychiatric Association (APA) 2013 grouped Autism spectrum disorder (ASD) as a single umbrella category, replacing the four different subtypes in DSM-IV-TR, which includes the autistic disorder, Asperger syndrome, childhood disintegrative disorder, and pervasive developmental disorder—not otherwise specified (PDD-NOS) [2]. ASD is a range of spectrum from mild to severely debilitating neurodevelopment disorder that typically presents before the age of 3 [1]. In Malaysia, it is estimated that there are approximately 12,800 cases of autism, and 1 out of 600 children in Malaysia are affected by ASD [3]. The prevalence of ASD has been increasing significantly over the past 20 years, which may be a consequence of a change in diagnostic criteria, policy, and practice changes, and increased awareness of the disorder [4].

Many studies have reported that parents who have children with ASD are at risk of psychological stress, anxiety, depression, and poor health compared to having children with other disabilities [5,6,7]. Parenting outcomes were influenced by ASD severity, behavioural problems related to ASD, communication skills, adaptation skills, and system support such as access to child services [8,9]. The families may experience financial strains, time pressures, marital conflict, social isolation, increased care time, reduced parental self-efficacy, inadequate support services, ongoing child advocacy, and uncertainty about the child’s future as stressors increase [10,11]. Good family wellbeing creates a bi-directional effect; the positive cycle can reduce the child’s problematic behaviour and increase positive response to the therapeutic invention. Conversely, impaired family functioning may result in adverse child outcomes [11,12].

Previous research exploring the experiences of families of children with disabilities living in rural areas is insufficient [13]. Parents in these areas have different cultural beliefs that have influenced their understanding of ASD as well as their decision-making [14] and accounted for inadequate knowledge and higher level of stigma among parents and in society [15]. The lack of ASD-related facilities in rural and suburban areas could lead to delayed assessment, diagnosis, and therapy to the children.

However, recent research has shown that, although some families are at risk of having multiple challenges, many families respond positively and adapt to this stress [2,9,16]. There are four common categories of coping strategies used by parents of children with ASD: (1) active-avoidance (e.g., substance use, self-indulgence, and emotion sensation); (2) focused issues (e.g., planning, problem-solving, and seeking appropriate social support); (3) positive coping (e.g., use of humour, positive reframing, and acceptance); and (4) religious/denial (e.g., using religion or spirituality, pretending the problem does not exist) [17]. Many previous studies have shown that over time the family responses may vary from maladjustment to healthy adaptation or vice versa over time as a result of changes in types of stressors or challenges [8,18]. Resultantly, current literature has moved away from the single cause and effect relationship between parental stress and pathology and has highlighted families’ successful adaptation and normality.

Therefore, the Resilience Model of Family Stress, Adjustment, and Adaptation was used as a theoretical framework for investigating the coping mechanisms of parents with children having ASD in Northeast Malaysia [19]. The resilience model is a strength-based model that has evolved from the theory of family stress. The focus of the resilience model is on family resilience or their ability to maintain a healthy balance despite adversity. The model has been used in many families’ stress, strengths, and outcomes studies of changes in family life, such as gains and losses, strains and transitions, and acute and chronic illnesses. The model helps to explain one’s ability to recover or adjust or even triumph over life-related stressors and changes [20]. The Resilience Model of Family Stress, Adjustment, and Adaptation refers to conceptualized family resilience as two distinct but related processes or phases. The first process, adjustment, involves the influence of protective factors that facilitate the parent’s ability and efforts to maintain functioning and perform developmental tasks in the context of risk factors. The second process, adaptation, involves the influence of the recovery factors in promoting the ability of the parent to rebound and adapt in situations of family crisis. The resilience model focuses on two levels of interaction: (a) individual family systems: This is how the family deals internally with problems or difficulties between its members. (b) Family-to-community: ways in which the family externally handles difficulties or problems through interactions between the family and the community [21].

This study’s research question was how the parents cope with the challenges in nurturing a child with ASD. The parents’ experiences on challenges of having children with ASD have been published in another paper [22]. This study aimed to explore the coping strategies of parents of children with ASD in Northeast Malaysia through the Family Stress, Adjustment, and Adaptation Resilience Model. A greater understanding of the identified crisis and coping strategies will facilitate health care professionals and institutional agencies to provide better care and support for the maintenance of parental resilience [23].

## 2. Materials and Methods

This study explored the coping strategies of parents with children having ASD through a qualitative approach. It has been suggested that through qualitative research, a deep understanding of the experience of parents with disabled children can be gained. Therefore, phenomenology is employed to understand and describe a specific phenomenon in-depth and to reach the essence of the participants’ experience of the phenomenon [24]. Parents who were primary caregivers of children diagnosed with ASD and were able to converse in the local language were included in the study. We exclude parents who have psychiatry illness or are deaf, mute, or illiterate. All subjects gave their informed consent for inclusion before they participated in the study. The study was conducted in accordance with the Declaration of Helsinki, and the protocol was approved by the Ethics Committee of Universiti Sains Malaysia (USM/JEPeM/18050243).

### 2.1. Setting and Participants

The study was conducted in Northeast Malaysia. A total of 21 parents of 24 children (aged 18 and below) diagnosed with ASD were recruited. The parents have been purposefully selected through our key informants who were psychiatrists and therapists at the hospital rehabilitation centre. In addition, they were screened for study suitability and invited by these key informants through an invitation letter. When they agreed to participate, their contact number was obtained so that they could be contacted by phone by the first author. Those who confirmed their participation were scheduled for an appointment according to their preferences. From February 2019 to July 2019, 23 in-depth interviews (IDIs) were conducted. Saturation was achieved when there was a repetition of data on emerging key thematic elements of the phenomenon under study, and there was no enrolment afterward.

### 2.2. Procedure

After obtaining written consent, the first author conducted a face-to-face, in-depth interview (IDI). The interviews were held in the Malay dialect and recorded using two digital voice recorders. The interviews were conducted in convenient locations, mostly at the hospital consultation room during the child’s follow-up or therapy sessions. The interviews lasted between 1 and 2 h. The participants were informed that their participation was entirely voluntary, and they were free to withdraw if they felt uncomfortable during the interview.

The participants were asked to complete the sociodemographic details (Table 1) prior to the interview. The interview began with open-ended questions and then followed by questions on parents’ coping experiences based on the semi structured interview guide (Table 2). Subsequently, the conversation evolved according to the parents’ answers to the initial questions. At the end of the interview, the participants were asked to spend 30 min writing an essay on their challenges and how to cope with these challenges. This complementary approach was used to increase the reliability of interview data and to acquire valuable yet relevant insights into the experience of parents who may be missed during the interview. For each interview, field notes were written based on our interpretation, knowledge, and interaction with the participants during the interview sessions. This note helped us better understand the phenomenon. Interviews were transcribed verbatim by either the first author or the hired transcriber.

We conducted the pilot study, supervised by one of the authors that expert in qualitative study (RDM) to ensure the suitability and comprehensibility of the semi-structured interview guide and to provide the same set of important questions with a detailed account of the parents’ experience. We included data from two of the pilot interviews in the analysis because of the rich data obtained. We invited these parents to join the formal study and subjected them to a second interview and an additional procedure after a week to ensure in-depth discussion and documentation of their account from their life experience.

## 3. Data Analysis

All the transcriptions and data from the transcripts entered NVivo for data analysis. Thematic analysis was employed in this study. First, the three researchers (W.N., L.H.Y. and M.M.Z.) critically re-read the first five transcripts to familiarize themselves with parents’ comprehensive meaning and perspectives. The first author then created a preliminary list of nodes in the NVivo^®^, and we coded the transcripts. We initially analysed all transcripts individually before considering the identified themes together as a whole to form an overall group analysis and organized them into interconnected hierarchies (that is, themes, subthemes, and categories). Then we set a meeting (W.N., L.H.Y., M.M.Z. and R.D.M.) to agree on the codes. This triangulation process helped to reduce inter-rater difference and came out with the appropriate codes that could be used throughout the data analysis process continued by W.N. To increase trustworthiness and check for coding accuracy, the research supervisors (L.H.Y. and M.M.Z.) reviewed the preliminary units of the code of meaning for all interview transcripts. They compared data analysis and themes against a consistency check, resolving any differences through discussion. Additionally, RDM, an expert qualitative researcher, after first reviewing the coding units for five transcripts and the themes/subjects for the overall sample, provided general comments and suggestions. After completing the data analysis, we once again made a meeting to reach a consensus within the research team, agreeing on the final codes and the themes, subthemes, and categories. We emailed all the transcripts and final emergent themes back to all parents for validation and confirmation within 2 weeks. None of them disagreed with these reports.

## 4. Results

A total of 21 parents of 24 children with ASD were included in this study. The parents’ mean age was 38 years (range = 29–54 years) with 8 fathers and 15 mothers that participated in this study. One mother had 5-year-old twins with ASD while another mother, all three of her children had ASD. The age of the children ranged from 2 to 14 years. The demographics of the parents are presented in Table 1. The analysis yielded three main themes: acceptance and positive outlook, reaching for helping hands, and understanding autism and finding its solution. Each of the parents interacted with each other to improve the adaptation of the ASD children’s families and ensure good well-being.

### 4.1. Acceptance and Positive Outlook

Acceptance of a child’s condition becomes an essential part of the life of parents whose children have ASD. Parents’ acceptance has an impact not only on their perception and well-being but also on their child. Therefore, acceptance was one of the most crucial elements of parent coping strategies observed in this study. Most parents feel that the key to well-being is to accept their children’s condition first, and the rest will follow. Acceptance also helps parents cope with their caregiving tasks.

Sixteen parents reported feeling anxious and sad about their children’s future upon learning about their ASD diagnosis. However, their visit to the psychiatric clinic made them realize that there were other less-fortunate people. As a result, they adjusted their thoughts and beliefs about their children’s condition and future. In addition, they became more positive and optimistic about adapting to a new life. P13 shared a turning point on her child’s condition.

“Of course, I was sad, but when I went to the hospital for my son’s therapy, I saw that other children were in a much worse condition like had to be wheelchair bounded. I thought I would have to be thankful because my son is physically healthy.”

Having a strong faith in God or religion helps family accept and overcome any ASD-related adversities. It gives meaning to their sacrifices. The parents explained how having and bringing up children with ASD strengthened their spiritual faith, which made them strong to overcome all the challenges they faced. Even though some parents initially felt sad about this fate, they later realized that God had given them a child with ASD not because of fate or past sins or misdeeds but because they had been specially chosen by God to take care of and raise such a special child. P19, father of two kids, told us, “We have always been grateful to have him. We have been very positive about this. Allah has chosen us to take care of this special child. We’re the one chosen.”

The parents also believed that their child with ASD was a gift from God, a source of good fortune for their families, and a key to heaven. It was a gift from God that needed to be patiently cherished. “Everyone’s goal is heaven. My son will definitely enter heaven. My hope is that he will bring me and the whole family to heaven as well.” (P9, businessman)

Families become resilient when they accept the condition and actively pursue solutions to their problems, look positively beyond the challenges they are facing, and focus on making the best of the options available to them. There was no time to despair and be in grief for a long time.

“We must be grateful. If we continue to grieve, the problem will never end. Over time, we will overcome this, we will accept his condition.”

(P14, gym trainer)

“Do not be ashamed of having an autistic child. We have got to accept them for who they are. We have got to treat them. Just spend more time with them. If we do not treat them, who else will?”

(P3, online seller)

Once the parents are able to accept their children’s condition, the others will follow. Their acceptance and positivity will make others, particularly the partners/spouses, other children, extended family members, and society, understand and accept this condition.

“When we have accepted our son’s condition, we are more open to telling others about our son’s condition, some people will understand and start offering support. Well, it works for me.”

(P17, research assistant)

Through acceptance, parents found a positive attitude in their lives, leading to both mental and life adaptation. Most of the parents (90%) made changes in terms of their priorities, particularly in family planning, job criteria, and daily schedules. P8 commented:

“I purposely requested to be a counselor instead of a special officer for my daughter’s sake…No matter how busy I am, I’m going to make sure I’m coming for her therapy.”

Parents had also adapted to their daily routines. They knew that their lives could no longer be spontaneous; they had to properly plan their lives and focus on the needs of their children with ASD. Maintaining family routines was crucial to preserve family cohesion and create a predictable environment for children with ASD struggling with the changes and the new situations. Half of the parents described continuous anticipation of the worst outcome and were always prepared to deal with it. The parents always planned their activities to deal with unforeseen behaviours. P20 said: “I know that he will throw a tantrum when he is hungry. So, I always bring food supply when we’re on vacation”.

### 4.2. Reaching for Helping Hands

Being a parent to a child with ASD can be emotionally and physically challenging, especially when dealing with the child’s idiosyncratic behaviours, sensory problems, and lack of communication. All parents identified social, emotional, government, and network support systems as the most critical support for addressing these challenges. The parents reported that family is their main pillar of strength to help them overcome the difficulties in their lives when raising a child with ASD. Family togetherness in dealing with caregiving routines helps relieve stress among parents. P14 shared:

“When my eldest son, daughter, and my husband are around, we’re going to do everything together, bathe him, feed him, wash him when he’s pooping.”

Some of the parents had a very supportive and understanding partner/spouse to help them overcome this hardship. Five parents shared their experience of being comforted by their partners whenever they lost motivation to take care of their children. P11 always expressed her stressful emotion to her husband. Similarly, P17 said: “When I was studying, my husband helped me a lot to take care of our son.” Their partners/spouses had helped them take a brief “me time” and take a break from the responsibility of caring for their child with ASD. Of the 21 parents, four believed that a short break from the routine caring for children with ASD would maintain their well-being and help them cope better. One of the parents said,

“During school holidays, when my husband is around, he’s going to take care of our son so I can go somewhere for a short vacation.”

(P10, housewife)

Others received support from extended family members, such as parents, siblings, employers, and even the local community. The supports they received were shared responsibility for caring, financial and emotional support, and ensuring the physical safety of the child with ASD.

“I did not make much money. Luckily, my father is helping me with milk, diapers, and clothes for my daughter. Sometimes when I cannot pick her up because of work, he is going to help me pick her up in the kindergarten.”

(P2, driver)

“My boss trusts me to arrange my own schedule without compromising my job. It makes it easier for me to come for the follow-up of my daughter.”

(P8, counselor)

The parents also appreciated the social support they received from close friends, technologically advanced social networks, and parents’ support community. It is a platform for acquiring relevant information on autism and the provision of emotional support and care advice, which are essential for their coping strategies.

“I always participate in the programs related to autism such as ‘Cakna Autism Kelantan’. We always make family gatherings. It’s a way for us to ventilate our emotions, share opinions, and get advice.”

(P9, businessman)

The parents received assistance from the government through a ‘person with different ability’ “*orang kelainan upaya* (OKU)” card. The ‘OKU’ card gives children with ASD many benefits, such as accessibility to special education/schools, lower hospital fees, parking lots, and many more.

“It’s really good in terms of facilities. The government has given him access to a lot of facilities through this ‘OKU’ card. The ‘OKU’ card benefits him, which is why they apply for it.”

(P20, physiotherapist)

### 4.3. Understanding Autism and Finding Its Solutions

This theme illustrates how parents manage their children with ASD using various methods, for example, both professional and alternative approaches. The parents’ goal in these actions was to improve the children’s capabilities and potential and prepare them for the future. Many parents (76%) began to educate themselves and the family about autism and its treatment. The parents reported making various attempts to gain resourceful information from close families working in the medical field, other parents, teachers, and healthcare providers. Subsequently, the parents shared their earnest attempt to understand this condition through in-depth searching on the Internet, Facebook, and books. It is a strategy that has convenient purposes but might also lessen the parents’ feeling of powerlessness.

“I didn’t know anything about autism before. Zero! After my son had autism, I did an in-depth reading on the Internet. I just know about autism and finally understand it. Now, I am more open minded to this.”

(P21, housewife)

Parents had tried their best and consistently followed all the intervention programs planned by the healthcare professional in hospitals and private therapeutic services, with the hope of a bright future for their children and a strong desire for their independence. All parents indicated that there had been an improvement in their children’s abilities over time. P1 had excitedly quoted:

“I am always looking forward to his therapy. A lot of new things and tips I have learned from doctors and therapists about my son’s therapy, which I can perform at home. I have seen a lot of improvement in him. He had poor eye contact before, but now he can focus. I’m so happy.”

(P1, housewife)

Some of the parents lowered their expectations on their children’s capabilities. The parents also learned to appreciate their children’s little achievement. They found that this coping strategy could ameliorate the effects of autism on them and help uphold their sense of hope.

“Although I spend a lot of money on private therapy, I am satisfied because he showed a lot of positive changes. Even though he is just able to hold the pencil right, it is a significant improvement for me.”

(P17, research assistant)

Finally, the parents’ cultural belief influences how parents cope with and handle this disorder. Aside from complying with professional therapies and advice, some parents (33 percent) tried using alternative methods, such as seeing traditional healers and Islamic Complementary Medicine (e.g, *Darul Syifa*), to optimally help their children. Parents made efforts to ensure that the child is “healed” or reduced in the severity of the condition.

“I met an *ustaz* (Islamic Healer). I requested for remedial water for my son so that he will be more behaved and less hyperactive. At least we tried our best, right?”

(P13, housewife)

## 5. Discussion

This study explored the experience of coping in the process of achieving resilience among parents of children with ASD in Northeast Malaysia. The analysis of the parent’s interviews in this study revealed dynamic mechanisms for becoming resilient in the Malay context. The three themes identified were acceptance and positive outlook, reaching for helping hands, and understanding autism and finding its solutions.

In this study, the Resiliency Model of Family Stress, Adjustment, and Adaptation was used as a theoretical framework to describe how the parents developed protective coping mechanisms to help them build and maintain resilience.

Despite the many challenges in raising children with ASD, families survive through a variety of coping strategies. Over time, the parents realized and learned that their perception of their children’s condition would have an impact on the outcome or consequences. In this study, we found that most parents were grateful and positively appreciative of this condition from being a crisis or stressor to a challenge and began to accept their children’s condition [1]. Therefore, parents changed their life priorities and adjusted their routines and life plans to best suit their children’s condition. They were empowered to be more vigilant in raising their children. It is an essential initial step toward adapting to the challenges of caring for children with ASD. As a result, the findings of our study are similar to a lot of research on coping strategies among parents who have children with ASD [2,3,4,5].

Social support appears to be a coping strategy that alleviates the stress felt by parents and improves their well-being and positive attitude toward their children with ASD [6,7]. This study demonstrated that a supportive environment and a high level of family cohesion can help parents manage their children with ASD. In this study, the parents reported that the family members had a high level of patience and were determined to work together to take good care of the child with ASD. The parents also reported how communication within the family improved since they had this special child. They have always felt a sense of relief, knowing that they can rely on their spouse when things become hard. The parents were able to ventilate their stress to their spouse and take a short time away from the child with ASD. Shared responsibilities contributed to better adaptation in these families [8,9]. Family cohesion also extended beyond the nuclear to the extended family. The parents and every member of the family were able to make a meaningful contribution to the life changes accompanying the child’s upbringing, which, in turn, has increased resilience and improved adaptation.

Aside from family support, this study demonstrated that social support has also been provided by local communities, autism societies, and government. The parents were surrounded by a neighborhood that was very supportive, understanding, and helpful in ensuring the child’s physical safety. Even though some who lack awareness of ASD may have shown unpleasant reactions, the local communities accepted them without any judgment. It gave them a sense of security and helped them build resilience. Similarly, the support received from the autism communities, either the parents’ groups or the social network, significantly helped these parents manage these children with ASD. It helped them gain knowledge about autism and obtain advice to improve self-efficacy, enabling them to feel a high level of confidence and less stress in parenting children with ASD [10,11]. Ilias and Liaw [5] reported disparities in the quality and accessibility of resources provided by the government. However, the parents in this study still received support from the government in terms of OKU card. The OKU card provides benefits to children with ASD in so many ways and guarantees their future. This study supports the findings by Ilias and Cornish [12], indicating that society and government support also plays a crucial role in building resilience.

Previous research has explained how spiritual and religious beliefs have influenced parents’ understanding and response to their children with ASD [2,9,16]. Religious beliefs have helped them make positive interpretations of having a child with ASD and given them support and encouragement during their difficult times. In the same way, this study demonstrated that having strong faith in God changed their belief and perception of this condition [4]. Strong faith encourages them to accept the child’s condition and to always be patient and tolerant at any time [17]. They treasure this child as a precious gift from God. They believed that they were the chosen one who had the opportunity for a better afterlife [18]. By putting all hopes in the hands of God, they felt closer to God and at ease, and they found resilience in their lives.

Parents found that gaining knowledge about autism is a fundamental aspect that would help them cope with this hardship [19]. Having a child with ASD encouraged parents to find out as much as possible about all the aspects of this condition, the treatments, and the services available to ensure that the child receives the most appropriate care. By seeking information and training, parents became acquainted with their child’s disorder and treatment [1]. They acquired knowledge from a variety of resources, including close families and friends; professional staff such as doctors, therapists, and teachers; and the Internet [20]. The Internet is an authoritative source of information for parents. They found that the information they had acquired from the lived experience of other ASD parents on Facebook had guided them in dealing with their children with ASD in their daily lives [8]. Similarly, the knowledge and advice obtained from professional staff, in particular, about the methods of behavioural modification, can help parents cope with ASD in their children [11].

In this study, the parents adapted problem-oriented strategies for coping with their children’s condition [21]. Over time, the parents began to fully understand the extent of their child’s disability and limitations. They articulated their efforts to arrange professional and non-professional treatments (e.g., traditional healers and complementary medicine) for their child and to develop strategies for their functional abilities [11,16,20]. The parents’ objective in these activities was to optimize the children’s capabilities and potential of their children as well as to prepare them for the future. The parents were committed to all therapy sessions to make the child better. They realized that having high expectations and wanting the child to be completely normal are unrealistic and have increased their stress levels. Therefore, parents have learned that having realistic goals and lowering their expectations will help them accept the child’s condition and achieve increased sensitivity, increased confidence and assertiveness, greater opportunities to learn, and a feeling of strength in the face of adversity. Parents have learned to appreciate and cherish little improvement in their children. In addition, they found that their lives could be happier and more meaningful by doing so. The health care professionals could use these study findings to create a module, course, pamphlet, or therapy session for parents on coping strategies in handling children with ASD. For example, to conduct a therapy session for parents on adapting their children that are newly diagnosed with ASD by accepting and having a positive outlook. Health care professionals could also organize a course for parents on how and when to search for help from various sources and support groups and to create a module or pamphlet on parents’ education to understand more on autism and find its solution.

The findings of this study are limited by the number of participants representing each ethnic group, which was inadequate and lacked involvement from fathers. However, it displayed the true ethnic proportions in this area, where 98% are Malays, and most of the main carers for children were their mothers. Another area that should have been emphasized is the socioeconomic background of the carers, which could be an important factor in their coping mechanism to having children with autism. Another limitation is that the structured interview guide might influence the direction of the responses and overlooked any negative coping mechanisms from the parents. We recommend including other ethnic groups and fathers as well as to have a wider structured interview guide to gain better understanding of the adaptation process of autism on the families.

## 6. Conclusions

This study discussed the value of using the Resiliency Model of Family Stress, Adjustment, and Adaptation; a theoretical approach; and qualitative methods to describe and define the unique coping strategies of parents who have children with ASD. This study demonstrated that despite a glimpse of the daily stress experienced by the parents, most of them have been able to adjust, cope well, and often grow up to meet the needs of these children. Finally, the identified factors can be incorporated into intervention programs and help guide specialized institutions in providing more comprehensive information and support for families caring for children with ASD. This study will help the health care professionals to provide better care and support to the parents by educating and counselling parents on the coping mechanism and adaptation methods in nurturing their children with ASD.

## Figures and Tables

**Table 1:** Socio-demographics of parents with ASD children (*n* = 21).

Parent’s Id	Age	Sex	Ethnicity	Occupation	Marital Status	Age of Child (Year)	Age of Diagnosis (Year)
P1	41	Female	Malay	Housewife	Married	3	1.5
P2	43	Male	Malay	Driver	Divorced	4	2.5
P3	37	Female	Malay	Housewife	Married	3	3
P4	38	Female	Malay	Housewife	Married	7/5/2	4/2/2
P5	38	Male	Malay	Barber	Married	6	2.5
P6	41	Female	Siamese	Freelance Accountant	Married	5	4
P7	32	Female	Malay	Nurse Assistant	Married	4	2
P8	33	Male	Malay	Counsellor	Married	7	2
P9	39	Male	Malay	Businessman	Married	9	5
P10	36	Female	Malay	Housewife	Married	7	3
P11	35	Female	Malay	Housewife	Married	5/5	5
P12	38	Female	Chinese	Businesswoman	Married	9	3
P13	32	Female	Malay	Housewife	Married	7	4
P14	40	Female	Malay	Gym trainer	Married	6	1.5
P15	54	Female	Malay	Housewife	Married	14	3
P16	36	Female	Malay	Assistant Auditor	Married	7	5
P17	32	Female	Malay	Research Assistant	Married	5	2.5
P18	29	Female	Malay	Housewife	Married	7	4
P19	36	Male	Malay	Fruit seller	Married	8	2
P20	51	Male	Malay	Physiotherapist	Married	14	2
P21	31	Female	Malay	Housewife	Married	5	2

**Table 2:** The semi-structured interview guide.

Introduction		
This study concerns the lived challenges and coping mechanisms as a parent with an autism spectrum disorder child. I am interested in your experiences regarding challenges you faced as a parent, and I wish to know about how you deal with it. Before we go further, would you please tell me about yourself? I would like for you to share your experiences from the time prior to your child’s diagnosis to the present period. It would be beneficial for you to explain your thoughts and feelings, what it is like to parent a child with autism, your parenting styles, the challenges you experience, the types of needs you have, the available support or the support that was both available and unavailable, the help you received, ways that you were able to cope, and anything else that comes to mind.		
**Questions**	**Specific Questions**	**Probing**
What helps you cope with the challenges or difficulties as a parent?	Adjustment: How do these issues help you in coping with your child with ASD? What are the things you need to assist you as a parent? (previously, now, future) Adaptation: How has your coping mechanism changed over time? What makes things easier for you?	Passive coping/denial Avoidance and isolation Empowerment & redirecting the energy Religion and spiritual Acceptance and acknowledgment Support system What do you think about your coping style before/now? How do you think you might cope with your child in the future?
**Ending**		
Is there anything you would like to add before we end (advice, improvement suggestions, etc.)? Thank you to the participants.		

## Data Availability

Data is contained within the article.
